# Benchmarking RCGAu on the Noiseless BBOB Testbed

**DOI:** 10.1155/2015/734957

**Published:** 2015-03-29

**Authors:** Babatunde A. Sawyerr, Aderemi O. Adewumi, M. Montaz Ali

**Affiliations:** ^1^School of Mathematics, Statistics and Computer Science, College of Agriculture, Engineering and Science, University of KwaZulu-Natal, Westville, South Africa; ^2^Department of Computer Sciences, Faculty of Science, University of Lagos, Lagos, Nigeria; ^3^School of Computational and Applied Mathematics, Faculty of Science and TCSE, Faculty of Engineering and Built Environment, University of the Witwatersrand, Johannesburg, South Africa

## Abstract

RCGAu is a hybrid real-coded genetic algorithm with “*uniform random direction*” search mechanism. The *uniform random direction* search mechanism enhances the local search capability of RCGA. In this paper, RCGAu was tested on the BBOB-2013 noiseless testbed using restarts till a maximum number of function evaluations (#FEs) of 10^5^ × *D* are reached, where *D* is the dimension of the function search space. RCGAu was able to solve several test functions in the low search dimensions of 2 and 3 to the desired accuracy of 10^8^. Although RCGAu found it difficult in getting a solution with the desired accuracy 10^8^ for high conditioning and multimodal functions within the specified maximum #FEs, it was able to solve most of the test functions with dimensions up to 40 with lower precisions.

## 1. Introduction

The simple genetic algorithm (GA) introduced by Holland is a probabilistic algorithm based on the theory of natural selection by Charles Darwin. GA mimics the evolutionary process through the creation of variations in each generation and the survival of the fittest individuals through the blending of genetic traits. Individuals with genetic traits that increase their probability of survival will be given more opportunities to reproduce and their offspring will also profit from the heritable traits. Over the period of time these individuals will eventually dominate the population [1, 2].

GA consists of a set of potential solutions called chromosomes, a selection operator, a crossover operator, and a mutation operator. A chromosome is a string of zeros (0s) and ones (1s). It is a metaphor of the biological chromosome in living organisms. The zeros (0s) and ones (1s) are called genes. A gene is the transfer unit of heredity. It contains genetic traits or information that is passed on from a parent solution to its offspring. The selection operator selects solutions for mating based on the principle of “*survival of the fittest*.” The crossover operator generates new solution pairs called children by combining the genetic materials of the selected parents. The mutation operator is an exploratory operator that is applied, with low probability, to the population of chromosomes to sustain diversity. Without the mutation operator, GAs can easily fall into premature convergence [1, 3].

The simple GA was designed to work on binary strings and it is directly applicable to pseudoboolean objective functions. However, most real life problems are represented as continuous parameter optimization problems. A decoding function was designed to map the solutions from binary space to the real-valued space. This decoding process can become prohibitively expensive for binary string GAs especially when the problem dimension increases [1, 3]. To tackle this problem real-coded genetic algorithms were introduced [4].

Real-coded genetic algorithms (RCGAs) use real-valued vectors to represent individual solutions. Surveys show that several variants of RCGAs have been proposed and used to solve a wide range of real life optimization problems. Some recent examples can be found in [1, 4–10].

Over the last three decades, researchers have continuously improved the performance of RCGAs through hybridization. RCGAs have been hybridized with other optimizers such as Nelder-Mead algorithms [11], simplex method [12], quadratic approximation [13], and pattern search [14–16].

In this paper, a set of noiseless testbed from the black-box optimization benchmarking (BBOB) 2013 workshop is used to benchmark RCGAu, a hybrid real-coded genetic algorithm that consists of “*uniform random direction*” local search technique.

The RCGAu algorithm is presented in Section 2, Section 3 provides the CPU timing for the experiments, Section 4 presents the results and discussion, and finally Section 5 concludes the paper with some recommendations.

## 2. The RCGAu Algorithm

RCGAu is a hybrid RCGA with a simple derivative-free local search technique called “*uniform random direction*” local search method. The local search technique operates on all individuals after the mutation operator has been applied to the population of individuals.

The RCGAu used in this work is a modified version of the RCGAu used in [16, 17]. It consists of five major operators, namely, tournament selection, blend-*α* crossover, nonuniform mutation, uniform random direction local search method, and a stagnation alleviation mechanism.  Algorithm 1 shows the RCGAu algorithm.

The notations used in this paper are defined as follows.

*P*_*t*_ denotes the population of individual solutions *x*_*i*,*t*_ at time *t*, *N* is the size of *P*_*t*_, *σ*(*f*(*P*_*t*_)) represents the standard deviation of the fitness values *f*(*P*_*t*_) of all solutions *x*_*i*,*t*_, ∈*P*_*t*_, ${\hat{P}}_{t}$ is the mating pool containing the parent solutions, *C*_*t*_ is the population of offspring solutions obtained after applying crossover on the parents in ${\hat{P}}_{t}$, *p*_*c*_ is the crossover probability, *M*_*t*_ is the resultant population of solutions after applying mutation on *C*_*t*_, *p*_*m*_ is the mutation probability, and *Υ*_*t*_ is the population of solutions obtained after ulsearch has been applied to *M*_*t*_, where ulsearch denotes the uniform random direction local search. Also, *ϵ* = 10^−12^, a very small positive value [18].

The evolutionary process in Algorithm 1 starts by initializing *P*_*t*=0_ from the search space *X* ⊂ *ℜ* ^*n*^. The domain of *X* is defined by specifying upper (*u* ^*j*^) and lower (*l* ^*j*^) limits of each *j*th component of *x*; that is, *l* ^*j*^ ≤ *x* ^*j*^ ≤ *u* ^*j*^ and *l* ^*j*^, *u* ^*j*^ ∈ *ℜ*, *j* = 1,2,…, *n*. Next, the fitness value *f*(*x*_*i*,*t*_), ∀*x*_*i*,*t*_ ∈ *P*_0_, is calculated and the population diversity of *P*_*t*_ is measured by calculating the standard deviation *σ*(*f*(*P*_*t*_)) of *f*(*x*_*i*,*t*_).

If *σ*(*f*(*P*_*t*_)) ≤ *ϵ* and the global optimum has not been found, then 90% of *P*_*t*_ is refreshed with newly generated solutions using the function perturb (*P*_*t*_). *P*_*t*_ is refreshed by sorting the solutions according to their fitness values and preserving the top 10% of *P*_*t*_. The remaining 90% of *P*_*t*_ are replaced with uniformly generated random values from the interval [−4,4]^*D*^ and the resultant population; ${\hat{P}}_{t}=\left\{x_{1,t},x_{2,t},\ldots ,x_{m,t}\right\}$ is created. *m* is the size of the mating pool ${\hat{P}}_{t}$ and *m* ≤ *N*. If, on the other hand, *σ*(*f*(*P*_*t*_)) > *ϵ* then tournament selection is applied on *P*_*t*_ to create an equivalent mating pool ${\hat{P}}_{t}$.

The tournament selection scheme works by selecting *r* number of solutions uniformly at random from *P*_*t*_, where *r* is the tournament size and *r* < *N*. The selected *r* individuals are compared using their fitness values and the best individual is selected and assigned to ${\hat{P}}_{t}$. This procedure is repeated *m* times to populate ${\hat{P}}_{t}$.

After the mating pool has been created, blend-*α* crossover is applied to a pair of parent solutions (*x*_*i*,*t*_, *x*_*k*,*t*_) if a randomly generated number *τ* drawn uniformly from the interval [0,1] is greater than the specified crossover probability threshold *p*_*c*_. Blend-*α* crossover creates a pair of offspring (*c*_1,*t*_, *c*_2,*t*_) from the interval [min⁡⁡(*x*_*i*,*t*_^*j*^, *x*_*k*,*t*_^*j*^) − *α*∗*d* ^*j*^, max⁡⁡(*x*_*i*,*t*_^*j*^, *x*_*k*,*t*_^*j*^) + *α*∗*d* ^*j*^] as follows:

(1) c1,tj=min⁡⁡xi,tj,xk,tj−α∗dj,max⁡⁡xi,tj,xk,tj+α∗djc2,tj=min⁡⁡xi,tj,xk,tj−α∗dj,max⁡⁡xi,tj,xk,tj+α∗dj,

where (1 ≤ *k* ≤ *N*), *α* = 0.3 + 0.2 × *z*, *z* is a uniform random number drawn from the interval [0,1], and *d* ^*j*^ = |*x*_*i*,*t*_^*j*^ − *x*_*k*,*t*_^*j*^|. The new pair (*c*_1,*t*_, *c*_2,*t*_) is then copied to the set *C*_*t*_; otherwise the pair (*x*_*i*,*t*_, *x*_*k*,*t*_) is copied to *C*_*t*_.

Then the nonuniform mutation [4] is applied to the components of each member of *C*_*t*_ with probability, *p*_*m*_, as follows:

(2) $$\begin{array}{c} m_{i,t}^{j}=\left\{\begin{array}{cc} c_{i,t}^{j}+\Delta \left(t,u^{j}-c_{i,t}^{j}\right) & \text{if}\;u\leq 0.5,\\ c_{i,t}^{j}-\Delta \left(t,c_{i,t}^{j}-l^{j}\right) & \text{otherwise}, \end{array}\right. \end{array}$$

where *u* is a uniformly distributed random number in the interval [0,1]. *u* ^*j*^ and *l* ^*j*^ are the upper and lower boundaries of *x* ∈ *X*, respectively. The function Δ(*t*, *u* ^*j*^ − *c*_*i*,*t*_^*j*^) given below takes a value in the interval [0, *y*]:

(3) $$\begin{array}{c} \Delta \left(t,y\right)=y{\left(1-r^{\left(1-(t/T)\right)}\right)}^{\beta }, \end{array}$$

where *r* is a uniformly distributed random number in the interval [0,1], *T* is the maximum number of generations, and *β* is a parameter that determines the nonuniform strength of the mutation operator. The mutated individual *m*_*i*,*t*_ is then copied to the set *M*_*t*_; otherwise *c*_*i*,*t*_ is copied to *M*_*t*_.

Then ulsearch is applied on each solution *m*_*i*,*t*_ ∈ *M*_*t*_ with the aim of performing local searches around the neighborhood of each solution. ulsearch works by randomly selecting a solution *m*_*i*,*t*_ ∈ *M*_*t*_ and creating a trial point *y*_*i*,*t*_ using

(4) $$\begin{array}{c} y_{i,t}=m_{i,t}+\Delta_{t}U, \end{array}$$

where Δ_*t*_ is a step size parameter and *U* = (*U*_1_, *U*_2_,…, *U*_*n*_)^*T*^ is a directional cosine with random components

(5) $$\begin{array}{c} U_{j}=\frac{R_{j}}{{\left(R_{1}^{2}+\cdots +R_{n}^{2}\right)}^{1/2}},\;j=1,2,\ldots ,n, \end{array}$$

Where  *R*_*j*_ ~ Unif([−1,1]). There are cases when the components of the trial point *y*_*i*,*t*_ = (*y*_*i*,*t*_^1^, *y*_*i*,*t*_^2^,…, *y*_*i*,*t*_^*n*^) generated by (4) fall outside the search space *X* during the search. In these cases, the components of *y*_*i*,*t*_ are regenerated using

(6) $$\begin{array}{c} y_{i,t}^{j}=\left\{\begin{array}{cc} m_{i,t}^{j}+\lambda \left(u^{j}-m_{i,t}^{j}\right), & \text{if}\;y_{i,t}^{j}>u^{j}\\ m_{i,t}^{j}+\lambda \left(m_{i,t}^{j}-l^{j}\right), & \text{if}\;y_{i,t}^{j}<l^{j}, \end{array}\right. \end{array}$$

where *λ* ~ Unif([0,1]) and *m*_*i*,*t*_^*j*^ is the corresponding component of the randomly selected solution *m*_*i*,*t*_ ∈ *M*_*t*_.

The step size parameter, Δ_*t*_, is initialized at time *t* = 0 according to [15, 16] by

(7) $$\begin{array}{c} \Delta_{0}=\tau \times \max\left\{u^{j}-l^{j}\;|\;j=1,2,\ldots ,n\right\}, \end{array}$$

where *τ* ∈ [0,1]. The idea of using (7) to generate the initial step length is to accelerate the search by starting with a suitably large step size to quickly traverse the search space and as the search progresses the step size is adaptively adjusted at the end of each generation, *t*, by

(8) $$\begin{array}{c} \Delta_{t+1}=\frac{1}{K}\overset{K}{\underset{i=1}{\sum }}\gamma^{i}, \end{array}$$

where *K* is the number of Euclidean distances {*γ* ^1^, *γ* ^2^,…, *γ* ^*K*^} between *K* nearest points to the mean $\bar{x}$ and $\bar{x}$ of a set of randomly selected distinct points *Ω* = {*x*_1_, *x*_2_,…, *x*_*q*_} ⊂ *P*_*t*_.

After the trial point *y*_*i*,*t*_ ∈ *Υ* has been created, it is evaluated and compared with *m*_*i*,*t*_. If *y*_*i*,*t*_ < *m*_*i*,*t*_, then *y*_*i*,*t*_ ∈ *Υ* is used to replace *m*_*i*,*t*_ ∈ *M*_*t*_; otherwise the search direction is changed by changing the sign of the step length. The new step length is used to recalculate a new trial point. After a new trial point has been recalculated and evaluated, it is used to replace *m*_*i*,*t*_ ∈ *M*_*t*_ with *y*_*i*,*t*_, if *y*_*i*,*t*_ < *m*_*i*,*t*_; otherwise *m*_*i*,*t*_ ∈ *M*_*t*_ is retained.

At the end of ulsearch, *P*_*t*_ is updated with *M*_*t*_ to form *P*_*t*+1_ and elitism is used to replace the worst point in *P*_*t*+1_ with the best point in *P*_*t*_ because the generational model is the replacement strategy adopted in this work [19].

## 3. Experimental Procedure and Parameter Settings

The experimental setup was carried out according to [20] on the benchmark functions provided in [21, 22]. Two independent restart strategies were used to restart RCGAu whenever *P*_*t*_ stagnates or when the maximum number of generations is exceeded and *f*_target_ is not found. For each restart strategy, the experiment is reinitialized with an initial population *P*_0_ which is uniformly and randomly sampled from the search space [−4,4]^*D*^ [6, 18].

Two stopping conditions used for the restart strategies are as follows.

- A test for stagnation is carried out to check if the best solution obtained so far did not vary by more than 10^−12^ during the last (50 + 25 × *D*) generations as in [6].
- A test is carried out to check if the maximum number of generations is satisfied and *f*_target_ is not found.

The parameters used for RCGAu on all functions are

- population size = min⁡⁡(100,10 × *D*), where *D* is the problem dimension;
- maximum number of evaluations #FEs = 10^5^ × *D*;
- tournament size *r* = 3;
- crossover rate *p*_*c*_ = 0.8;
- mutation rate *p*_*m*_ = 0.15;
- nonuniformity factor for the mutation *β* = 15;
- elitism *E* = 1;
- crafting effort CrE = 0 [20].

## 4. CPU Timing Experiment

The CPU timing experiment was conducted for RCGAu using the same independent restart strategies on the function *f*_8_ for a duration of 30 seconds on an AMD Turion (tm) II Ultra Dual-Core Mobile *M*620 CPU processor, running at 2.50 GHz under a 32-bit Microsoft Windows 7 Professional service pack 1 with 2.75 GB RAM usable and Matlab 7.10  (*R*2010*a*).

The time per function evaluation was 2.5, 2.6, 2.9, 3.0, 3.2, and 3.5 times 10^−4^ seconds for RCGAu in dimensions 2, 3, 5,10, 20, and 40, respectively.

## 5. Results

**Figure 1:**
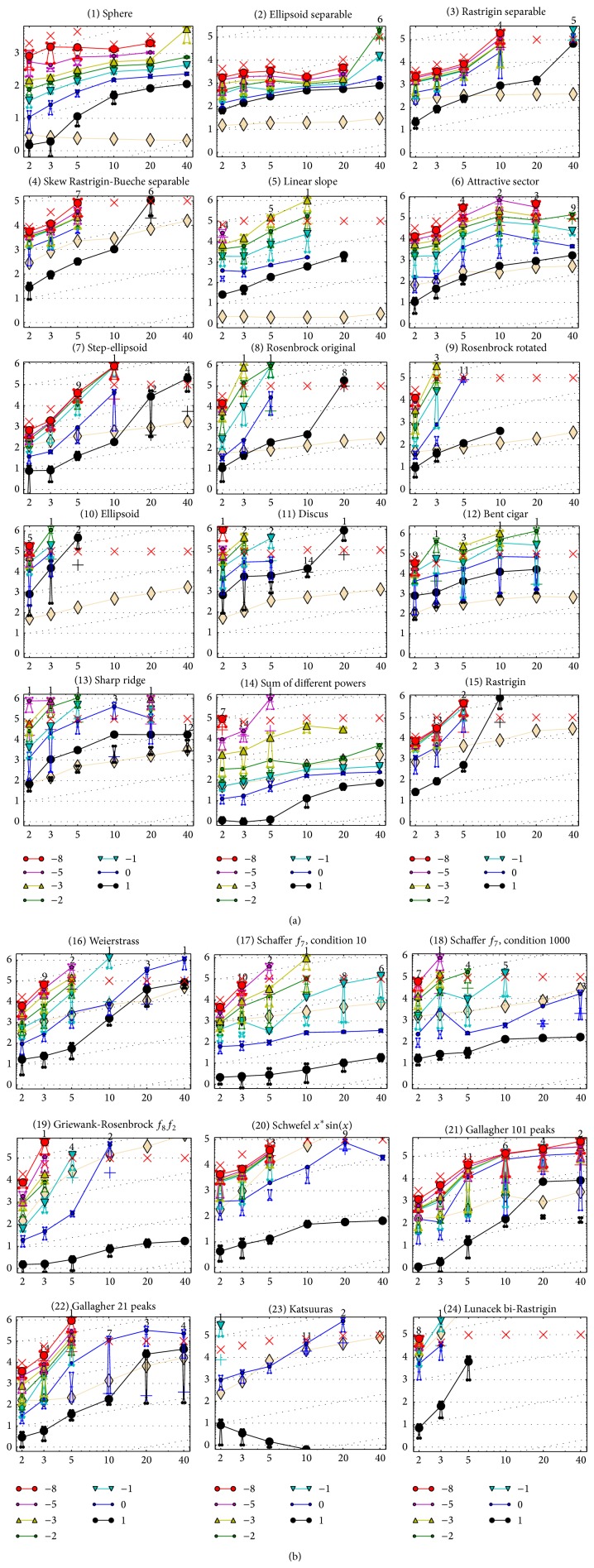
Expected number of *f*-evaluations (ERT, lines) to reach *f*_opt_ + Δ*f*; median number of *f*-evaluations (+) to reach the most difficult target that was reached not always but at least once; maximum number of *f*-evaluations in any trial (×); interquartile range with median (notched boxes) of simulated run lengths to reach *f*_opt_ + Δ*f*; all values are divided by dimension and plotted as log⁡_10_⁡ values versus dimension. Also, Δ*f* = 10^{1,0,−1,−2,−3,−5,−8}^ are shown. Numbers above ERT-symbols (if appearing) indicate the number of trials reaching the respective targets. The light thick line with diamonds indicates the respective best results from BBOB-2009 for Δ*f* = 10^−8^. Horizontal lines mean linear scaling and slanted grid lines depict quadratic scaling.

**Figure 2:**
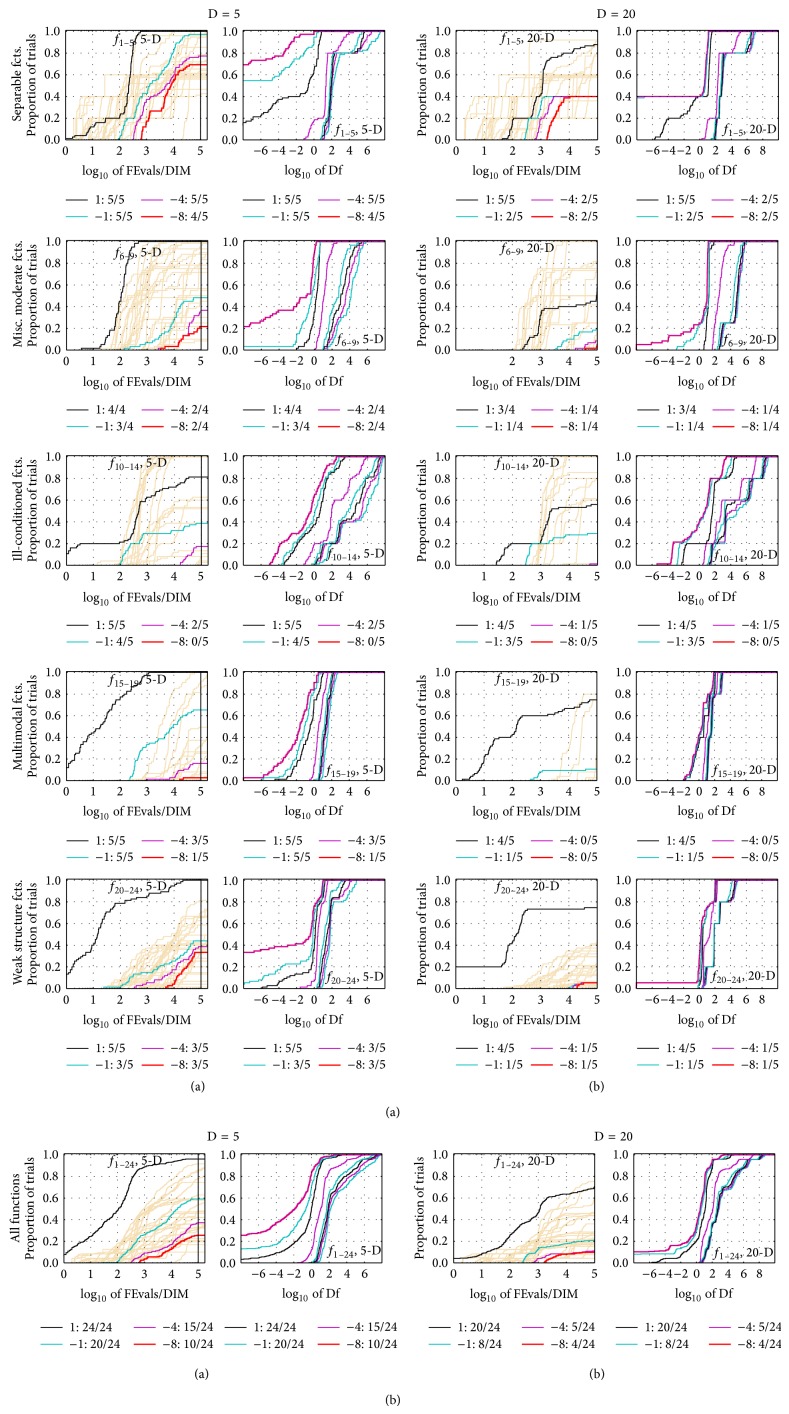
Empirical cumulative distribution functions (ECDF), plotting the fraction of trials with an outcome not larger than the respective values on the *x*-axis. Left subplots: ECDF of the number of function evaluations (FEvals) divided by search space dimension *D*, to fall below *f*_opt_ + Δ*f* with Δ*f* = 10^*k*^, where *k* is the first value in the legend. The thick red line represents the most difficult target value *f*_opt_ + 10^−8^. Legends indicate for each target the number of functions that were solved in at least one trial within the displayed budget. Right subplots: ECDF of the best achieved Δ*f* for running times of 0.5*D*, 1.2*D*, 3*D*, 10*D*, 100*D*, 1000*D*,… function evaluations (from right to left cycling cyan-magenta-black…) and final Δ*f*-value (red), where Δ*f* and Df denote the difference to the optimal function value. Light brown lines in the background show ECDF for the most difficult target of all algorithms benchmarked during BBOB-2009.

**Figure 3:**
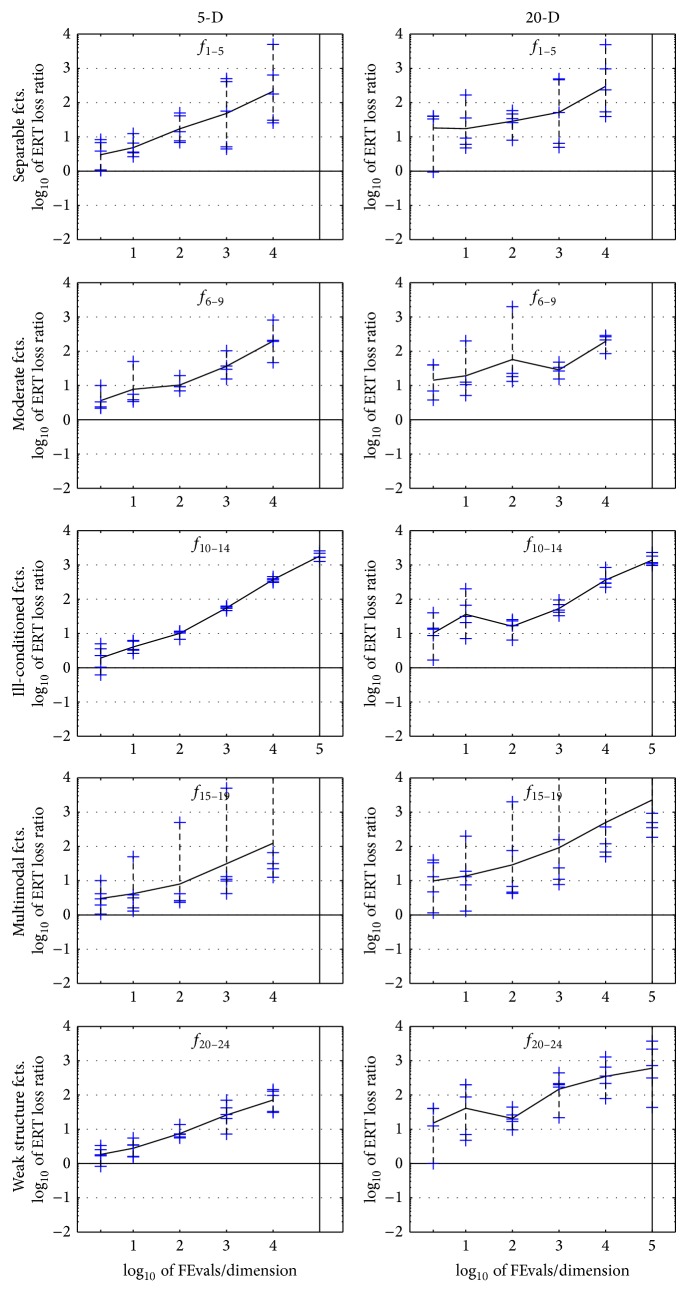
ERT loss ratios (see Table 2 for details). Each cross (+) represents a single function and the line is the geometric mean.

The results of the empirical experiments conducted on RCGAu according to [20] on the benchmark functions given in [21, 22] are presented in Figures 1, 2, and 3 and in Tables 1 and 2.

Figure 1 shows the performance of RCGAu on all the noiseless problems with the dimensions 2, 3, 5, 10, 20, and 40. RCGAu was able to solve many test functions in the low search dimensions of 2 and 3 to the desired accuracy of 10^8^. It is able to solve most test functions with dimensions up to 40 at lowest precision of 10^1^.

Although RCGAu found it difficult in getting a solution with the desired accuracy 10^8^ for high conditioning and multimodal functions within the specified maximum #FEs it was able to solve *f*_21_ with dimensions up to 40, *f*_1_ and *f*_2_ with dimensions up to 20, *f*_3_ and *f*_7_ with dimensions up to 10, and *f*_4_, *f*_6_, *f*_15_, *f*_20_, and *f*_22_ with dimensions up to 5.

In Figure 2, the left subplot graphically illustrates the empirical cumulative distribution function (ECDF) of the number of function evaluations divided by the dimension of the search space, while the right subplot shows the ECDF of the best achieved Δ*f*. This figure graphically shows the performance of RCGAu in terms of function evaluation.

Table 1 presents the performance of RCGAu in terms of the expected running time (ERT). This measure estimates the run time of RCGAu by using the number of function evaluations divided by the best ERT measured during BBOB 2009 workshop. This benchmark shows that RCGAu needs some improvement in terms of performance.

## 6. Conclusion

The performance of RCGAu on the suite of noiseless black-box optimization testbed has been average on a number of problems but it has excelled in solving functions *f*_1_, *f*_2_, *f*_3_, *f*_7_, and *f*_21_. Studies have currently been carried out to find out why RCGAs do not efficiently solve highly conditioned problems. Further modifications to RCGAs are needed to exploit the full strength of evolutionary processes.

## Figures and Tables

**Algorithm 1:**
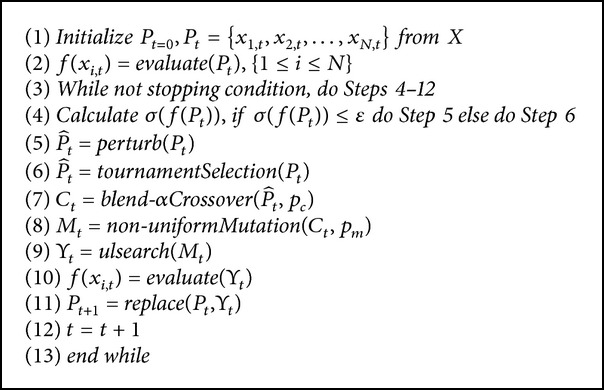
The RCGAu Algorithm.

**Table 1:** Expected running time (ERT in number of function evaluations) divided by the best ERT measured during BBOB-2009. The ERT and, in braces, as dispersion measure, the half difference between 90 and 10 percentile of bootstrapped run lengths appear in the second row of each cell, the best ERT in the first. The different target Δ*f*-values are shown in the top row. #succ is the number of trials that reached the (final) target *f*_opt_ + 10^−8^.

5D									20D								
Δ*f*	1*e* + 1	1*e* + 0	1*e* − 1	1*e* − 2	1*e* − 3	1*e* − 5	1*e* − 7	#succ	Δ*f*	1*e* + 1	1*e* + 0	1*e* − 1	1*e* − 2	1*e* − 3	1*e* − 5	1*e* − 7	#succ
**f**_1_	11	12	12	12	12	12	12	15/15	**f**_1_	43	43	43	43	43	43	43	15/15
	5.2 (6)	27 (15)	56 (15)	91 (22)	131 (29)	319 (193)	580 (800)	15/15		39 (7)	90 (17)	148 (19)	218 (28)	294 (36)	506 (112)	787 (173)	15/15

**f**_2_	83	87	88	89	90	92	94	15/15	**f**_2_	385	386	387	388	390	391	393	15/15
	16 (3)	21 (6)	29 (10)	71 (118)	79 (118)	112 (180)	155 (176)	15/15		29 (5)	39 (8)	50 (8)	66 (17)	81 (30)	128 (41)	189 (78)	15/15

**f**_3_	716	1622	1637	1642	1646	1650	1654	15/15	**f**_3_	5066	7626	7635	7637	7643	7646	7651	15/15
	1.8 (1)	7.8 (9)	13 (15)	13 (15)	16 (16)	21 (20)	24 (19)	15/15		6.6 (6)	∞	∞	∞	∞	∞	*∞1.1e6 *	0/15

**f**_4_	809	1633	1688	1758	1817	1886	1903	15/15	**f**_4_	4722	7628	7666	7686	7700	7758	1.4e5	9/15
	2.0 (0.9)	26 (26)	62 (83)	60 (79)	60 (63)	99 (109)	129 (134)	7/15		459 (473)	∞	∞	∞	∞	∞	*∞1.1e6 *	0/15

**f**_5_	10	10	10	10	10	10	10	15/15	**f**_5_	41	41	41	41	41	41	41	15/15
	93 (20)	359 (112)	3404 (3910)	15683 (18516)	76807 (77456)	∞	*∞2.7e5 *	0/15		1075 (273)	∞	∞	∞	∞	∞	*∞1.2e6 *	0/15

**f**_6_	114	214	281	404	580	1038	1332	15/15	**f**_6_	1296	2343	3413	4255	5220	6728	8409	15/15
	6.5 (6)	95 (217)	263 (443)	432 (351)	470 (459)	552 (489)	749 (685)	4/15		14 (3)	75 (28)	277 (325)	365 (470)	465 (474)	971 (1056)	1110 (1193)	3/15

**f**_7_	24	324	1171	1451	1572	1572	1597	15/15	**f**_7_	1351	4274	9503	16523	16524	16524	16969	15/15
	8.4 (6)	14 (22)	58 (89)	98 (114)	126 (139)	126 (133)	125 (134)	9/15		402 (507)	∞	∞	∞	∞	∞	*∞1.0e6 *	0/15

**f**_8_	73	273	336	372	391	410	422	15/15	**f**_8_	2039	3871	4040	4148	4219	4371	4484	15/15
	13 (4)	517 (732)	13503 (15418)	12258 (13199)	∞	∞	*∞2.8e5 *	0/15		1796 (1474)	∞	∞	∞	∞	∞	*∞2.0e6 *	0/15

**f**_9_	35	127	214	263	300	335	369	15/15	**f**_9_	1716	3102	3277	3379	3455	3594	3727	15/15
	17 (7)	3920 (3354)	∞	∞	∞	∞	*∞5.0e5 *	0/15		∞	∞	∞	∞	∞	∞	*∞2.0e6 *	0/15

**f**_10_	349	500	574	607	626	829	880	15/15	**f**_10_	7413	8661	10735	13641	14920	17073	17476	15/15
	6725 (7986)	∞	∞	∞	∞	∞	*∞3.1e5 *	0/15		∞	∞	∞	∞	∞	∞	*∞2.0e6 *	0/15

**f**_11_	143	202	763	977	1177	1467	1673	15/15	**f**_11_	1002	2228	6278	8586	9762	12285	14831	15/15
	205 (236)	698 (864)	2442 (2575)	∞	∞	∞	*∞2.6e5 *	0/15		17715 (19470)	∞	∞	∞	∞	∞	*∞1.1e6 *	0/15

**f**_12_	108	268	371	413	461	1303	1494	15/15	**f**_12_	1042	1938	2740	3156	4140	12407	13827	15/15
	201 (576)	303 (585)	490 (704)	1392 (1453)	2674 (2913)	∞	*∞2.7e5 *	0/15		320 (961)	706 (1035)	2024 (2557)	8893 (9507)	∞	∞	*∞2.0e6 *	0/15

**f**_13_	132	195	250	319	1310	1752	2255	15/15	**f**_13_	652	2021	2751	3507	18749	24455	30201	15/15
	115 (216)	2009 (2341)	9467 (10306)	16356 (17378)	∞	∞	*∞3.3e5 *	0/15		529 (907)	1072 (1477)	2052 (2407)	5467 (5911)	1026 (1134)	792 (852)	*∞1.2e6 *	0/15

**f**_14_	10	41	58	90	139	251	476	15/15	**f**_14_	75	239	304	451	932	1648	15661	15/15
	0.62 (0.6)	5.9 (5)	13 (5)	50 (23)	406 (473)	17736 (19499)	*∞3.1e5 *	0/15		13 (6)	18 (3)	24 (5)	53 (29)	612 (196)	∞	*∞2.0e6 *	0/15

**f**_15_	511	9310	19369	19743	20073	20769	21359	14/15	**f**_15_	30378	1.5*e*5	3.1*e*5	3.2*e*5	3.2*e*5	4.5*e*5	4.6*e*5	15/15
	5.1 (4)	50 (60)	81 (91)	120 (151)	118 (142)	115 (142)	114 (130)	2/15		∞	∞	∞	∞	∞	∞	*∞1.2e6 *	0/15

**f**_16_	120	612	2662	10163	10449	11644	12095	15/15	**f**_16_	1384	27265	77015	1.4*e*5	1.9*e*5	2.0*e*5	2.2*e*5	15/15
	2.3 (2)	25 (7)	50 (79)	38 (49)	69 (80)	196 (217)	*∞2.9e5 *	0/15		569 (695)	227 (268)	∞	∞	∞	∞	*∞1.3e6 *	0/15

**f**_17_	5.2	215	899	2861	3669	6351	7934	15/15	**f**_17_	63	1030	4005	12242	30677	56288	80472	15/15
	2.7 (3)	2.3 (0.8)	1.8 (0.6)	27 (33)	47 (57)	308 (353)	*∞2.6e5 *	0/15		3.2 (3)	6.0 (2)	294 (397)	∞	∞	∞	*∞1.1e6 *	0/15

**f**_18_	103	378	3968	8451	9280	10905	12469	15/15	**f**_18_	621	3972	19561	28555	67569	1.3*e*5	1.5*e*5	15/15
	1.5 (1)	3.3 (0.8)	11 (11)	101 (106)	∞	∞	*∞2.6e5 *	0/15		4.7 (2)	22 (3)	∞	∞	∞	∞	*∞1.1e6 *	0/15

**f**_19_	1	1	242	1.0*e*5	1.2*e*5	1.2*e*5	1.2*e*5	15/15	**f**_19_	1	1	3.4*e*5	4.7*e*6	6.2*e*6	6.7*e*6	6.7*e*6	15/15
	13 (15)	1532 (999)	2682 (3069)	∞	∞	∞	*∞1.8e5 *	0/15		277 (139)	∞	∞	∞	∞	∞	*∞1.1e6 *	0/15

**f**_20_	16	851	38111	51362	54470	54861	55313	14/15	**f**_20_	82	46150	3.1*e*6	5.5*e*6	5.5*e*6	5.6*e*6	5.6*e*6	14/15
	4.2 (3)	12 (21)	3.4 (3)	2.7 (3)	2.7 (3)	3.0 (3)	3.4 (3)	13/15		15 (3)	33 (33)	∞	∞	∞	∞	*∞1.2e6 *	0/15

**f**_21_	41	1157	1674	1692	1705	1729	1757	14/15	**f**_21_	561	6541	14103	14318	14643	15567	17589	15/15
	1.8 (2)	50 (60)	64 (106)	71 (101)	72 (100)	96 (121)	109 (116)	11/15		261 (4)	338 (435)	304 (365)	300 (362)	295 (344)	280 (317)	250 (301)	4/15

**f**_22_	71	386	938	980	1008	1040	1068	14/15	**f**_22_	467	5580	23491	24163	24948	26847	1.3*e*5	12/15
	2.5 (2)	113 (258)	364 (426)	545 (684)	754 (889)	1337 (1548)	4297 (4718)	1/15		1036 (2143)	1139 (1385)	∞	∞	∞	∞	*∞1.2e6 *	0/15

**f**_23_	3.0	518	14249	27890	31654	33030	34256	15/15	**f**_23_	3.2	1614	67457	3.7*e*5	4.9*e*5	8.1*e*5	8.4*e*5	15/15
	2.4 (1)	37 (42)	∞	∞	∞	∞	*∞1.7e5 *	0/15		1.7 (2)	5701 (6238)	∞	∞	∞	∞	*∞1.1e6 *	0/15

**f**_24_	1622	2.2*e*5	6.4*e*6	9.6*e*6	9.6*e*6	1.3*e*7	1.3*e*7	3/15	**f**_24_	1.3*e*6	7.5*e*6	5.2*e*7	5.2*e*7	5.2*e*7	5.2*e*7	5.2*e*7	3/15
	20 (27)	∞	∞	∞	∞	∞	*∞1.8e5 *	0/15		∞	∞	∞	∞	∞	∞	*∞1.1e6 *	0/15

**Table 2:** ERT loss ratio versus the budget (both in number of *f*-evaluations divided by dimension). The target value *f*_*t*_ for a given budget FEvals is the best target *f*-value reached within the budget by the given algorithm. Shown is the ERT of the given algorithm divided by best ERT seen in GECCO-BBOB-2009 for the target *f*_*t*_, or, if the best algorithm reached a better target within the budget, the budget divided by the best ERT. Line: geometric mean. Box-Whisker error bar: 25–75%-ile with median (box), 10–90%-ile (caps), and minimum and maximum ERT loss ratio (points). The vertical line gives the maximal number of function evaluations in a single trial in this function subset. See also Figure 3 for results on each function subgroup.

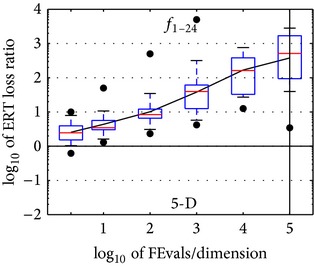			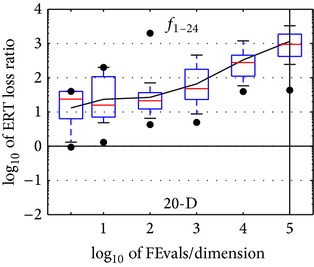			

#FEs/*D*	*f*_1_ –*f*_24_ in 5-D, maxFE/*D* = 100018					
	best	10%	25%	**med**	75%	90%

2	0.62	1.0	1.4	2.5	4.0	8.5
10	1.3	1.6	2.9	3.5	5.8	16
100	2.3	2.6	6.4	8.4	13	42
1*e*3	4.2	5.0	12	40	62	4.2*e*2
1*e*4	13	25	32	1.6*e*2	3.9*e*2	1.2*e*3
1*e*5	3.4	35	83	5.1*e*2	1.7*e*3	3.0*e*3
1*e*6	3.4	35	1.4*e*2	8.6*e*2	1.2*e*4	2.2*e*4
RL_US_/*D*	3*e*4	4*e*4	5*e*4	5*e*4	7*e*4	1*e*5

#FEs/*D*	*f*_1_ –*f*_24_ in 20-D, maxFE/*D* = 100004					
	best	10%	25%	**med**	75%	90%

2	0.94	1.1	5.8	24	40	40
10	1.3	4.8	7.0	16	1.3*e*2	2.0*e*2
100	4.3	6.2	11	21	39	2.7*e*2
1*e*3	5.0	7.6	23	48	1.8*e*2	4.7*e*2
1*e*4	39	53	1.0*e*2	2.8*e*2	5.2*e*2	1.7*e*3
1*e*5	43	2.3*e*2	4.0*e*2	9.4*e*2	1.9*e*3	8.3*e*3
1*e*6	2.4*e*2	4.2*e*2	9.9*e*2	3.9*e*3	1.1*e*4	6.8*e*4
RL_US_/*D*	4*e*4	5*e*4	5*e*4	6*e*4	1*e*5	1*e*5
